# Application of FTIR Method for the Assessment of Immobilization of Active Substances in the Matrix of Biomedical Materials

**DOI:** 10.3390/ma12182972

**Published:** 2019-09-13

**Authors:** Dorota Kowalczuk, Monika Pitucha

**Affiliations:** 1 Chair and Department of Medicinal Chemistry, Faculty of Pharmacy with Division of Medical Analytics, the Medical University of Lublin, 20-090 Lublin, Poland; 2 Independent Radiopharmacy Unit, Faculty of Pharmacy with Division of Medical Analytics, the Medical University of Lublin, 20-093 Lublin, Poland

**Keywords:** FTIR analysis, biomaterial characterization, biomaterial modification, immobilization

## Abstract

Background: The purpose of the study was to demonstrate the usefulness of the Fourier transform infrared spectroscopy (FTIR) method for the evaluation of the modification process of biomaterials with the participation of active substances. Methods: Modified catheter samples were prepared by activating the matrix with an acid, iodine, or bromine, and then immobilizing the active molecules. To carry out the modification process, the Fourier transform infrared-attenuated total reflectance (FTIR-ATR) method was used. Results: FTIR analysis indicated the presence of the immobilized substances in the catheter matrix and site-specific reactions. Conclusion: We surmise that the infrared spectroscopic technique is an ideal tool for the assessment of the drug immobilization and the changes occurring in the course of the modification process.

## 1. Introduction

Fourier transform infrared spectroscopy (FTIR) is a universal analytical tool for evaluation of a wide range of materials [1,2], especially for identification of unknown materials [3]. The FTIR technique has been used to identify pure substances [4], mixtures [5], impurities [4,6], and compositions of various materials [7,8]. It has also been helpful in the elucidation of different processes concerning plenty of compounds or materials. Papers available in the specialist literature report application of infrared spectroscopy for the analysis of structural changes [9] as well as for monitoring production (bioproduction) processes [10,11,12]. From the viewpoint of pharmaceutical sciences, the study of substance content in formulations, the study of substance release from formulations, and other kinetic processes, including substance penetration or distribution, are important applications of mid infrared spectroscopy [13,14].

FTIR spectroscopy has also been helpful in performing more advanced analyses aimed at the characterization of artistic materials [14] and disease diagnosis [15,16]. With regard to its medical applications, the use of FTIR for protein structure characterization based on the amide linkage of the peptides is of great importance as well [16]. FTIR spectroscopy is a pivotal technique for characterization of polymeric and biopolymeric materials [17,18]. The FTIR technology has proved to be useful in identification and characterization of homopolymers, copolymers, or polymer composite, and it has been successfully applied in the evaluation of polymerization process, characterization of the polymer structure, polymer surface, polymer degradation, and polymer modification [17,19,20]. More common applications of FTIR methodology include: Quality verification of materials; analysis of thin films and coatings [21]; decomposition of polymers and other materials, often through thermogravimetry combined with FTIR [22,23] and mass spectrometry; microanalysis of materials to identify contaminants; monitoring of emissions; and failure analysis [24,25,26]. The speed of FTIR analysis makes it particularly useful in screening applications, while its sensitivity allows many advanced research applications. Thus, infrared spectroscopy is a valuable technique for analysis of pharmaceuticals, polymers and plastics, food, environment, and falsified materials, including medicines [27,28,29]. Advance techniques of FTIR are as follows: Fourier transform infrared-attenuated total reflectance (FTIR-ATR), Fourier transform infrared-photo acoustic spectroscopy (FTIR-PAS), Fourier transform infrared imaging spectroscopy (FTIR spectrometer combined with an optical microscope, FTIR-Microscopy), Fourier transform infrared microspectroscopy, and Fourier transform infrared nanospectroscopy (nano-FTIR spectroscopy), which achieves nanoscale-level spatial resolution by combining IR spectroscopy and scattering-type near-field scanning microscopy [1,2,9,10,29,30]. Infrared nanospectroscopy opens up new possibilities for chemical and structural analysis and quality control in various fields, ranging from materials’ sciences to biomedicine [30].

Over the past years, much attention has been focused on modification of biomaterials and their potential applications in medical implants or other biomedical devices. The advanced FTIR technologies are also considered very helpful in assessing material changes [31,32,33,34,35,36,37,38]. Therefore, it is not surprising that the universality of FTIR methods has often been reported by researchers [31,32,34]. Infrared spectroscopy has been widely used for characterization of materials (e.g., latex, silicone, polyurethane) subjected to different physicochemical treatment when better hydrophilicity, lubricity, haemocompatibility, biocompatibility, or antibacterial activity were aimed at [31,32,33,34,35]. Evaluation of the modification progress relied on comparing the surface spectra of the untreated and treated materials. Registration of the material sample spectra after each subsequent stage of modification enabled identification of even the smallest changes without the infrared spectra library searching [31,32,35].

A variety of material modifications related to the use of physical (plasma, microwave or UV radiation, electron beam irradiation), chemical (e.g., ozone oxidation, enzymatic oxidation, surface graft polymerization, site-specific reactions), or physicochemical treatment were monitored by means of infrared spectroscopic instrumentation [31,32,33,34,35,36,37,38].

A qualitative FTIR analysis is often the first step in the analytical process of a material. Fourier transform infrared spectroscopic method, however, provides a quantitative analysis as well. What is more, FTIR is a relatively cheap technique that might permit direct semi-quantitative measurements of various organic entities, including the adsorbed/immobilized compounds. As a result of using a standard calibration curve of known concentrations, such measurements become quantitative [39,40]. The advantages of the FTIR quantitative procedure include direct evaluation of the adsorbed/immobilized amount as well as exact measurement of compounds that are hard to quantify, since they do not absorb UV-visible light, or they require expensive analytical procedures [39].

The FTIR spectroscopy combined with chemometric analysis provides very rapid, accurate, and generic approaches to the characterization not only of chemical compounds and biomolecules, but also potential biomarkers and microorganisms, including microbial pathogens [41].

IR spectrochemical analysis in combination with the chemometric technique (for example partial least square discriminant analysis, partial least square regression) can also be applied as a new diagnostic tool in microbiological and medical diagnostics [42,43].

Vibrational spectroscopy, like FTIR and Raman spectroscopy, plays an important role in assessing the covalent and non-covalent functionalization of biomaterials obtained through the incorporation of various functionalities at the material matrix or surface, including immobilization of active compounds [44,45]. These complementary methods can be used for the discrimination between covalent and non-covalent immobilizations. The appearance of new functional groups in the FTIR spectra of functionalized materials may testify to covalent modifications, while the shift of spectral bands characteristic of the material or immobilized molecule may indicate non-covalent interactions (Van der Waals interactions, hydrogen bonds) [17,19,46,47].

In the previous studies, we used FTIR-ATR method for analysis of the multistep modification of biomaterials with the covalent immobilized molecules [46,47]. Continuing the modification research, we have developed a two-step method of non-covalent immobilization of the active substance in the material matrix. 

The aim of this study was to present some examples of biomaterial modification in order to point out the importance of FTIR spectroscopy in the evaluation of the presence of an active substance introduced to a biomaterial matrix, site-specific changes, as well as to elucidate the mechanism of immobilization. 

## 2. Materials and Methods

All chemicals used for the modification process of biomaterials were purchased from Sigma-Aldrich (Poznan, Poland), and POCH (Polish Chemical Reagents, Gliwice, Poland) companies. The modification process of biomaterials was performed using the selected patented procedures [48,49], adapted for the presented research. 

### 2.1. Preparation of P-2-CA-Treated Catheter Samples

The samples were prepared by immobilization of pyrrole-2-carbaldehyde (P-2-CA, from Sigma-Aldrich Sp. z o.o., Poznan, Poland), on commercial polyurethane intravenous catheters (BD Becton Dickinson, Franklin Lakes, NJ, USA). The catheters were cut into 1 cm long fragments and activated in an acid solution under gentle stirring at 50 °C. Then the samples were removed, rinsed several times with distilled water, and dried. In the next stage, the samples were placed in Eppendorf tubes, immersed in 1 mL of 2 mg mL^−1^ solution of pyrrole-2-carbaldehyde in dichloromethane, and incubated in closed tubes for 30 min, at 60 °C with stirring at 100 rpm, and then in open tubes to evaporate the solvent. Next, the catheters were removed, rinsed with water, and left to dry overnight.

### 2.2. Preparation of Sparfloxacin-Treated Catheter Samples

The samples were prepared by immobilization of sparfloxacin obtained from Sigma-Aldrich, on commercial latex catheters (Rusch, Germany). The catheters were cut into 0.5 cm long fragments and activated with 2% methanolic iodine solution for 1 h under gentle stirring at 40 °C. Next, the samples were removed, rinsed with distilled water, and dried. In the next stage, the samples were placed in Eppendorf tubes, immersed in 1 mL of 2 mg mL^−1^ solution of sparfloxacin and incubated in closed tubes for 12 h, at 60 °C with stirring at 100 rpm. Then the catheters were removed, rinsed with water, and left to dry overnight.

### 2.3. Preparation of Sethacridine-Treated Catheter Samples

The samples were prepared by immobilization of ethacridine lactate obtained from Sigma-Aldrich, on commercial polyurethane intravenous catheters (BD Becton Dickinson). The catheters were cut into 1 cm long fragments and activated with 1% methanolic bromine solution for 1 h under gentle stirring at 40 °C. Next, the samples were removed, rinsed with distilled water, and dried. In the next stage, the samples were placed in Eppendorf tubes, immersed in 1 mL of 2 mg mL^−1^ solution of ethacridine and incubated in closed tubes for 1 h at 60 °C with stirring at 100 rpm, and then in open tubes about 5 h. Then the catheters were removed, rinsed with water, and left to dry overnight.

### 2.4. Fourier Transform Infrared Spectroscopy (FTIR) Characterization

FTIR with transmittance mode was used to characterize the presence of specific chemical groups in the tested catheter samples. FTIR measurements were carried out on a Nicolet 6700 spectrometer (Thermo Fisher Scientific Inc., Warsaw, Poland) equipped with a deuterated triglycine sulfate detector (DTGS/KBr) and a versatile Attenuated Total Reflectance (ATR) sampling accessory with a diamond crystal plate. Spectra were recorded in the spectral range of 4000–600 cm^−1^ at 4 cm^−1^ spectral resolution, 2 sample gain, and 32 sample/background scans using OMNIC 8.1 computer software (Thermo Fisher Scientific Inc.).

## 3. Results and Discussion

We have been doing research into immobilization of active substances on the surface (or in the matrix) of biomaterials for many years. In our work, in order to assess the effectiveness of the modification process, we have used mainly Fourier transform infrared spectroscopy (FTIR). For this purpose, the FTIR spectra of the chemically treated and untreated samples were collected in the mid-infrared region using attenuated total reflection (ATR) sampling mode and then compared. In this paper, we present the selected results of the evaluation of the modified biomedical materials.

### 3.1. Surface Characterization of the Porphyrin-Coated Catheter

**Scheme 1:**
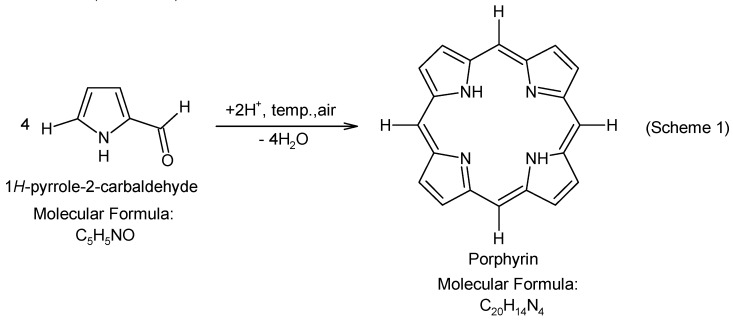
Synthesis of porphyrin.

**Figure 1:**
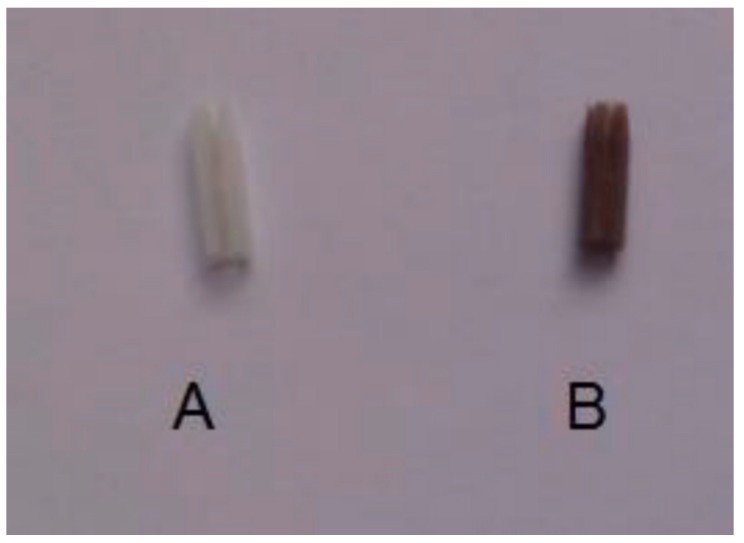
Catheter sample untreated (**A**) and porphyrin-coated (**B**).

Bacteria that form a heterogeneous and organized biofilm structure show increased resistance to antibiotics and they are therefore difficult to eradicate by means of a conventional antibiotic therapy. However, promising results have been obtained with the use of a photodynamic therapy (PDT) with cationic porphyrins as a photosensitizer. Studies have shown that the positive charge of cationic porphyrins determines the activity against Gram-positive and Gram-negative bacteria. It has been proved that PDT is highly useful in eliminating bacterial, fungal, protozoan, and viral infections [50,51,52]. PDT is a promising technology for the treatment of infectious diseases caused by antibiotic-resistant bacteria [52]. It can be used especially for the eradication of biofilms from the surface of catheters, prostheses, drains [53], and for the treatment of the localized infections [54]. Thus, photodynamic antimicrobial chemotherapy (PACT) may be an alternative to the conventional antibiotic therapy [50,52]. In PACT, the combination of a photosensitizer (light absorbing molecule) and light, in the presence of oxygen, results in the formation of cytotoxic free radicals (reactive oxygen species) that lead to the selective destruction of microbial cells [55]. Taking into account the effectiveness of PACT, we covered a catheter with a porphyrin photosensitizer with a view to prevention of biofilm formation on the catheter surface. The catheter was modified by activating its surface with a solution of nitric acid and then immobilization of pyrrole-2-carbaldehyde. In the acidic environment and in the presence of air, the tetrapyrrolic chain was formed [56,57] on the catheter surface (Scheme 1):

In the course of time, the surface of the catheter became reddish-violet suggesting the formation of porphyrin (Figure 1). 

**Scheme 2:**
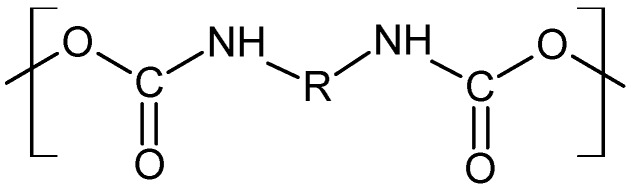
Organic unit typical of a urethane polymer.

**Figure 2:**
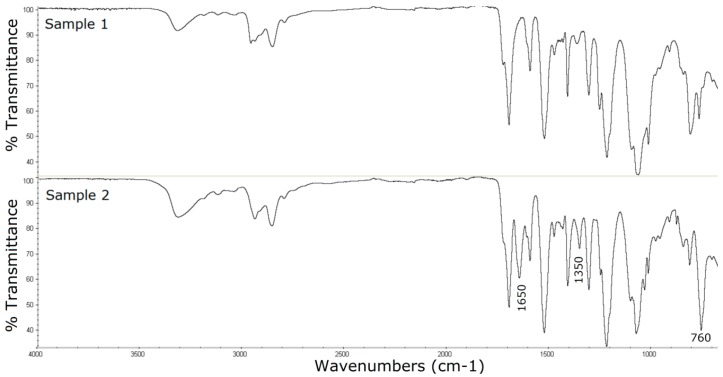
Fourier transform infrared spectroscopy (FTIR) spectra of the untreated polyurethane catheter (**Sample 1**) and P-2-CA-treated catheter with the formation of porphyrin (**Sample 2**).

The FTIR analysis (Figure 2) of the modified catheter surface confirmed our visual observations. The untreated polyurethane catheter (Sample 1) exhibited a typical distribution of absorption bands for this type of material. The vibrational band at 3330 cm^−1^ corresponds to N-H stretching vibrations. The peaks in the range from 3000 to 2800 cm^−1^ are connected with C-H asymmetrical and symmetrical stretching vibrations. The double peak in the region of 1680–1740 cm^−1^ (C=O stretching vibrations), the bands above 1500 cm^−1^ (probably N-H bending vibrations and C-N stretching vibrations), and the strong bands in the region of 1300–1000 cm^−1^ (asymmetrical and symmetrical O-C-O stretching vibrations) are associated with the bonds of urethane group (Scheme 2): 

Similar assessments of polyurethane based-materials have been presented earlier [58,59]. Besides, the available publications [59,60] report that the band at 1702 cm^−1^ is assigned to the hydrogen-bonded C=O (hydrogen bonding between C=O and N-H) of urethane linkage, while the peak at about 1720 cm^−1^ is related to free C=O group.

In the case of porphyrin FTIR spectrum, the main bands can be assigned according the published data. The bands at about 3300 cm^−1^ and 960 cm^−1^ are related to N-H bond stretching and bending frequencies of the pyrrole ring (Scheme 1), the zone of 3100–2900 cm^−1^ is assigned to C-H bond of the porphyrin ring (Scheme 1), the region of 1500–1680 cm^−1^, and the band at about 1350 cm^−1^ are assigned to C=C stretching and C=N stretching vibrations, respectively, while the band at about 760 cm^−1^ refers to the C-H bond bending vibrations [61].

The FTIR spectra of the unmodified and modified catheter are similar. However, several new porphyrin-specific peaks appear in the spectrum of the modified catheter, especially at 1650 cm^−1^ (C=C stretching vibrations), 1350 cm^−1^ (C=N stretching vibrations), and 760 cm^−1^ (=C-H bending vibrations)

### 3.2. Surface Characterization of the Catheter Coated with a Chemotherapeutic

**Scheme 3:**
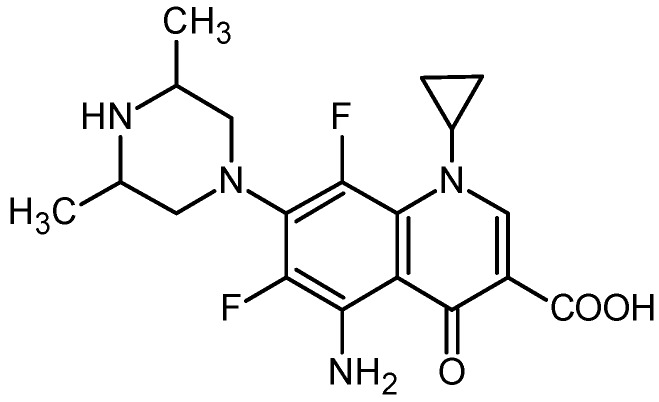
Structure of sparfloxacin.

In order to restrict the catheter-associated infections, it is recommended that the conventional antibiotic therapy should be applied, and the catheters should be impregnated with silver or antibiotics. Modification of biomaterials is the subject of many scientific studies. One of the strategies is incorporation of an antibacterial substance into the matrix of the biomaterial (not only to its surface) from which the substance could be gradually released (drug-eluting biomaterials). We performed a two-step modification by activating the latex catheter with the iodine solution and then incorporating the antimicrobial molecule from the fluoroquinolone group-sparfloxacin (Scheme 3):

This group of antibiotics was selected with regard to a broad spectrum of antimicrobial activity, good penetration properties, and hence their efficacy against the “young” and “old” biofilm and long-time persistence in the biofilm [62].

The presence of sparfloxacin on the surface of the biomaterial was confirmed by assessing the catheter surface at each stage of its modification using the FTIR method.

**Figure 3:**
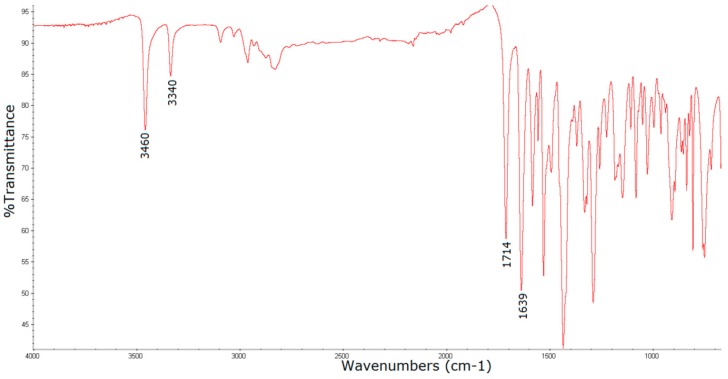
FTIR spectrum of sparfloxacin.

The FTIR spectrum of sparfloxacin shows many absorption bands (Figure 3). The most characteristic bands can be assigned to: Primary amine (νN-H in NH_2_) at 3460 cm^−1^ and 3340 cm^−1^, carbonyl (νC=O) in COOH at 1714 cm^−1^, carbonyl (νC=O) in 4-quinolone ring at 1639 cm^−1^, aromatic ring (νC=C) in the region of 1585–1400 cm^−1^ (with N-H in NH_2_), C-N bonding (νC-N in C-NH_2_), C-O bonding (νC-O in COOH) in the range of 1350–1260 cm^−1^,and C-F stretching vibrations in the range of 1250–1100 cm^−1^ [46].

**Figure 4:**
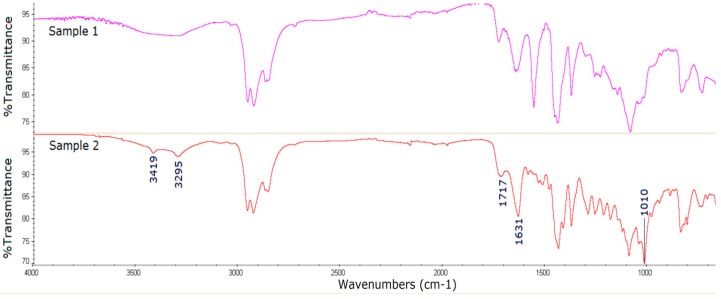
FTIR spectra of the iodine-activated latex catheter (**Sample 1**) and sparfloxacin-treated catheter (**Sample 2**).

The spectrum of the iodine-activated latex catheter (Figure 4, Sample 1) shows the absorbance bands characteristic of latex: C-H stretching vibrations (CH_3_ and CH groups) in the range of 3000–2850 cm^−1^, C=C stretching vibrations in the range of 1680–1600 cm^−1^, C-H blending vibrations, in-plane at about 1450–1350 cm^−1^ and out of plane at about 840 cm^−1^ [54], as well as the bands that appeared under the influence of iodine at about 1550 cm^−1^, 1050 cm^−1^, probably as a result of oxidation in the double bond region [63].

In the FTIR spectrum of the modified material (activated with iodine and treated with sparfloxacin) there are bands characteristic of sparfloxacin, which definitely confirms its presence: The intense bands at 3419 cm^−1^ and 3295 cm^−1^ associated with N-H asymmetric and symmetric stretching vibrations, the peaks at 1717 cm^−1^ and 1631 cm^−1^ corresponding to νC=O in COOH and to νC=O in 4-quinolone ring, respectively, and the drug peaks overlapping with the bands of the matrix corresponding to stretching vibrations of the aromatic ring and C-O, C-N, C-F bonds of sparfloxacin molecule in the range of 1450–1100 cm^−1^.

### 3.3. Surface Characterization of the Catheter Coated with an Antiseptic

Another strategy for the protection of the biomaterial against the formation of a bacterial biofilm is the introduction of an antiseptic into its matrix instead of an antibiotic.

**Scheme 4:**
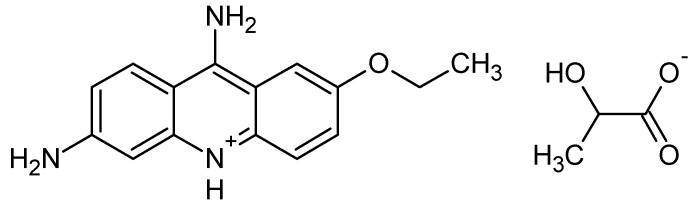
Structure of ethacridine lactate.

**Figure 5:**
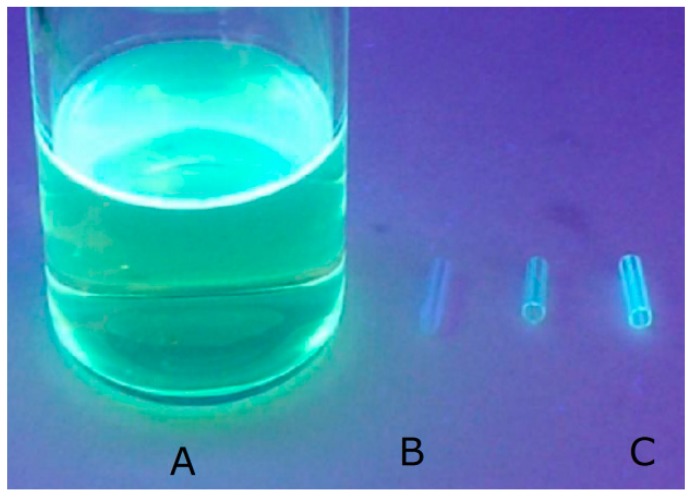
Ethacridine solution (**A**), the untreated catheter (**B**) and the ethacridine-treated catheter (**C**).

Thus, in a similar manner as before, polyurethane-based catheters were subjected to a two-stage modification. Initially, the polyurethane material was activated using a bromine solution and then treated with ethacridine lactate (Scheme 4):

The intense fluorescence of the catheter under UV light at 366 nm, similar to the fluorescence of ethacridine solution, may indicate the presence of ethacridine in the catheter matrix (Figure 5).

**Figure 6:**
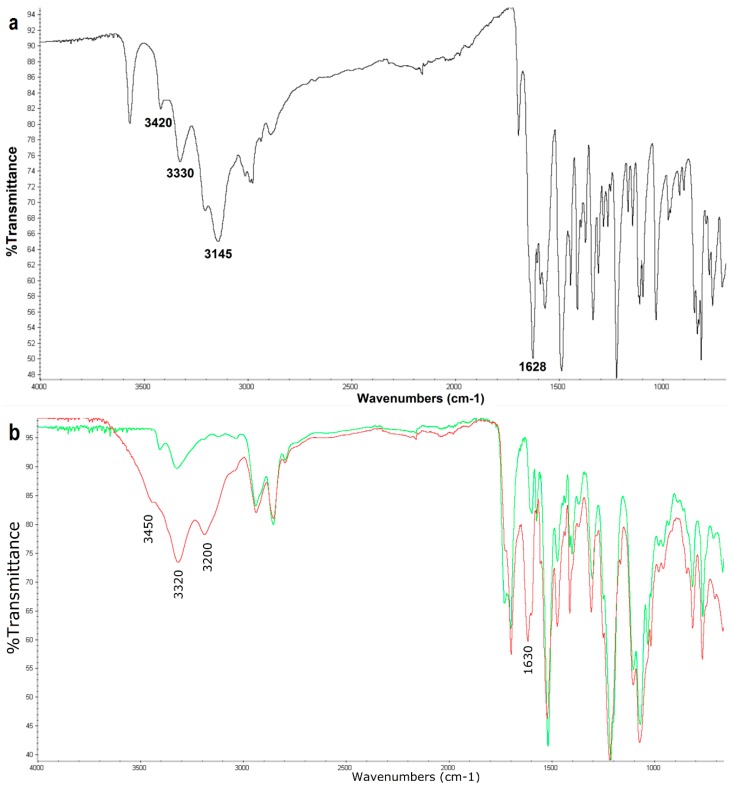
FTIR spectrum of ethacridine lactate (**a**); FTIR spectra of the bromine-treated catheter (green line) and the ethacridine-treated catheter (red line) (**b**).

The above observations confirmed the FTIR analysis of the modified catheter. The FTIR spectra obtained for the bromine-activated catheter and the ethacridine-treated catheter are shown in Figure 6.

The FTIR spectrum of ethacridine lactate [64] exhibits the bands in the region of 3500–3100 cm^−1^ which are assigned to N-H asymmetric and symmetric stretching vibrations of primary aromatic amine and hydrogen-bonded N-H stretching vibrations. The appearance of these bands in the FTIR spectra of the modified catheter located at about 3450, 3320, and 3200 cm^−1^, indicates the presence of ethacridine in the catheter matrix. Alongside this, the appearance of a relatively strong band at 1630 cm^−1^ corresponding to C=N stretching vibrations of the acridine ring can provide further evidence of the incorporation of ethacridine into the catheter matrix.

In the case of immobilization of sparfloxacin and ethacridine, no vibrational bands indicating a covalent binding of the drug with the formation of a new moiety were observed. Contrary to the previous studies, when sparfloxacin was covalently bound on a catheter coated with a heparin layer, we observed the appearance of an intense vibration band belonging to a new functional group formed as a result of linking the amino group of the drug with the carbonyl group of the oxidized heparin [46].

An increase in the band intensity or shifting to lower wavenumber values compared to pure substances (shift of 10–40 units) could suggest Van der Waals type interactions or hydrogen bonds between the introduced drug and matrix structure. The substances have probably been incorporated to the matrix by a process of activator-enhanced diffusion and/or by the changes in the polymer structure made by the activating agent which were observed in the spectrum of polyurethane polymer treated with iodine.

## 4. Conclusions

To sum up, our studies clearly indicate that the FTIR method with attenuated total reflection (ATR) is an important, indispensable, and effective tool for the assessment of the immobilization of active substances in the matrix of biomedical materials. Moreover, the fact that FIR-ATR is a relatively inexpensive, rapid, simple, and nondestructive technique, without sample pretreatment was of great importance in selecting the most appropriate experimental technique for the presented analyses. 

Three examples have been presented in order to illustrate the significant role of the combination of library searching focused on identification of characteristic spectral bands of the polymer, with the analysis of the registered spectra focused on identification of the functional groups of active compounds introduced to polymer matrix

Obtained modified biomaterials containing the immobilized, pharmacologically active substance are suggestive of potential applications in medicine to prevent the formation of bacterial biofilms that commonly trigger infections.
